# Are prosodic effects on sentence comprehension dependent on age?

**DOI:** 10.1590/2317-1782/20212021062

**Published:** 2023-03-03

**Authors:** Talita Fortunato-Tavares, Richard G. Schwartz, Claudia Regina Furquim de Andrade, Derek Houston, Klara Marton

**Affiliations:** 1 Speech-Language-Hearing Sciences Department, Lehman College, City University of New York - New York (NY), USA.; 2 Speech-Language-Hearing Sciences, The Graduate Center, City University of New York - New York (NY), USA.; 3 Departamento de Fisioterapia, Fonoaudiologia e Terapia Ocupacional, Faculdade de Medicina, Universidade de São Paulo – USP - São Paulo (SP), Brasil.; 4 The Ohio State University College of Medicine - Columbus (OH), USA.; 5 Communication Arts, Sciences and Disorders, Brooklyn College, City University of New York - New York (NY), USA.

**Keywords:** Child Language, Prosody, Syntax, Syntactic Ambiguity, Sentence Comprehension, Linguagem Infantil, Prosódia, Sintaxe, Ambiguidade Sintática, Compreensão de Frases

## Abstract

**Purpose:**

to investigate prosodic boundary effects on the comprehension of attachment ambiguities in Brazilian Portuguese and to test two hypotheses relying on the notion of boundary strength: the absolute boundary hypothesis (ABH) and the relative boundary hypothesis (RBH). Manipulations of prosodic structure influence how listeners interpret syntactically ambiguous sentences. However, the role of prosody in spoken language comprehension of sentences has received limited attention in languages other than English, particularly from a developmental perspective.

**Methods:**

Twenty-three adults and 15 children participated in a computerized sentence comprehension task involving syntactically ambiguous sentences. Each sentence was recorded in eight different prosodic forms with acoustic manipulations of F0, duration and pause varying the boundary size to reflect predictions of the ABH and RBH.

**Results:**

Children and adults differed in how prosody influenced their syntactic processing and children were significantly slower than adults. Results indicated that interpretation of sentences varied according to their prosodic forms.

**Conclusion:**

Neither the ABH or the RBH explained how children and adults who speak Brazilian Portuguese use prosodic boundaries to disambiguate sentences. There is evidence that the way prosodic boundaries influence disambiguation varies cross-linguistically.

## RESUMO

**Objetivo:**

investigar os efeitos de fronteiras prosódicas na compreensão de ambiguidades sintáticas no português brasileiro além de testar duas hipóteses baseadas na noção de intensidade de fronteira: a hipótese de fronteira absoluta (ABH) e a hipótese de fronteira relativa (RBH). Manipulações da estrutura prosódica influenciam como os ouvintes interpretam frases sintaticamente ambíguas. No entanto, o papel da prosódia na compreensão da linguagem oral tem recebido atenção limitada em línguas além do inglês, particularmente do ponto de vista do desenvolvimento.

**Método:**

Vinte e três adultos e 15 crianças participaram de uma tarefa computadorizada de compreensão de frases envolvendo frases sintaticamente ambíguas. Cada frase foi gravada em oito formas prosódicas diferentes com manipulações acústicas de F_0_, duração, e pausa, variando o tamanho da fronteira prosódica de modo a transparecer as previsões da ABH e RBH.

**Resultados:**

Crianças e adultos diferiram em como a prosódia influenciou o processamento sintático; as crianças foram significativamente mais lentas que os adultos. Os resultados indicaram que a interpretação das frases variou de acordo com suas formas prosódicas.

**Conclusão:**

Nenhuma das hipóteses (ABH ou RBH) explica como crianças e adultos falantes do Português brasileiro utilizam as fronteiras prosódicas para desambiguar frases. Há evidências de que a maneira com a qual os limites prosódicos influenciam a desambiguação de frases varia entre os idiomas.

## INTRODUCTION

Sentence comprehension depends on several factors: the lexical content at the word level, the structure at the syntactic level, and the prosody form in which it is delivered. Prosody plays an important role in spoken sentence comprehension and may influence syntactic interpretations^(1)^ affecting the resolution of syntactic ambiguities^(2-5)^. Two theoretical accounts aim to explain how prosody affects the resolution of syntactic ambiguities based on position and size of prosodic boundaries. The first, the Anti-attachment Hypothesis^(6)^ focuses on the effect of a single prosodic boundary immediately preceding the ambiguously attached phrase marked as B (see example [a] below): the absence of a boundary at B favors low attachment (only teachers have large bags), whereas the presence of a boundary at B favors high attachment (students and teachers have large bags). Carlson and colleagues argued that previous studies based on this hypothesis typically involved a larger boundary at B than the one marked at A below. They demonstrated in a series of comprehension studies with different syntactic constructions (e.g., relative clauses, conjunctions, and others) that the relative size (i.e. acoustic magnitude) of boundaries A and B has an effect on the interpretation of English-speaking adults^(7,8)^. This led to the subsequent Informative Boundary Hypothesis stating that prosodic boundaries interact with each other such that the effect of the boundary at B depends on the size of any earlier relevant boundary (e.g., A, on [a] below). When the boundary at A is larger than B, low attachment is favored; when boundary at B is bigger than A, high attachment is favored; when the two boundaries are equivalent, neither is favored.

- Students**_A_** and teachers**_B_** with large bags are at school.

Boundary size has been measured in previous studies by the ToBI (tones and break indices) coding system^(9)^. The ToBI is a prosodic annotation procedure that specifies the size of a boundary and distinguishes between a word boundary (0), an intermediate phrase boundary (ip) and an intonational phrase boundary (IPh). In general, IPh boundaries are accompanied by pauses of approximately 300 ms, ip boundaries contain pauses of approximately 100 ms, and 0 boundaries have no pauses. The same pattern is observed for F0 and duration, with IPhs involving more extreme changes than ips, which then in turn show more F0 and duration changes than 0 boundaries.

Snedeker and Casserly^(10)^ investigated how maintaining the size of the low boundary (B) constant while varying the size of the high boundary (A), changed sentence interpretation to an inverse of the Anti-Attachment Hypothesis. In the same study, they also sought to explore the influence of relative boundary size suggested in the Informative Boundary Hypothesis. The authors proposed two hypotheses related to boundary strength: the Absolute Boundary Hypothesis (ABH) and the Relative Boundary Hypothesis (RBH). The ABH relates to the Anti-Attachment Hypothesis and states that the absolute size of the prosodic boundary predicts syntactic attachment independent of the high boundary: a boundary in B favors high attachment and the absence of a boundary in B favors low attachment. In contrast, the RBH relies on predictions of the Informative Boundary Hypothesis and states that the *relative size* of the two boundaries (A and B) predicts attachment: a larger B than A boundary favors high attachment, a larger A than B boundary favors low attachment, and when the two are equivalent, neither attachment is favored (see Table 1 for predictions of ABH and RBH for each A,B boundary pair). Three boundary pairs have different predictions under the two hypotheses: IPh, ip has a low attachment prediction under the RBH and a high attachment prediction under the ABH; 0,0 and ip,ip have neutral predictions under the RBH and low and high attachment predictions respectively, under the ABH.

**Table 1:** Attachment Predictions for ABH and RBH according to relative size of boundaries on the A,B pair

**Boundary Pairs (A,B)**	**Relative Size of A and B**	**ABH Attachment Prediction**	**RBH Attachment Prediction**
0, 0	A=B	Low	Neutral
ip, ip	A=B	High	Neutral
0, ip	A<B	High	High
ip, IPh	A<B	High	High
0, IPh	A<B	High	High
ip, 0	A>B	Low	Low
IPh, 0	A>B	Low	Low
IPh, ip	A>B	High	Low

Caption: ABH = Absolute Boundary Hypothesis; RBH = Relative Boundary Hypothesis; IPh = intonational boundary; ip = intermediate boundary; 0 = null boundary

By adopting the ToBI system, Snedeker and Casserly^(10)^ developed specific predictions of the ABH and RBH that relate to the placement of prosodic boundary pairs. Under the ABH, sentences with a large boundary in B have higher probabilities of high attachment than sentences with small boundary B. Under this rationale, all predictions of the ABH are considered regardless of the size of the boundary in A. For the RBH, it is the relative size of boundary A compared to boundary B that matters. Sentences in which the boundary in B is larger than the boundary in A have higher probabilities of high attachment than sentences in which the boundary in B is equal to the boundary in A. In turn, sentences where A is equal to B have higher probabilities of high attachment than sentences in which the B boundary is smaller than the A boundary.

The unique predictions of ABH and RBH in English-speaking adults were tested^(10)^ and neither hypothesis alone was sufficient to account for the relation between prosodic phrasing and the attachment of an ambiguous phrase. However, the authors did find that the high boundary influenced interpretation when there was no boundary in B (0,0 > ip,0; 0,0 > IPh,0) as predicted by the RBH. Additionally, the high boundary influenced the interpretation of sentences that would be predicted to have neutral or low-attachment prosody based on the low boundary alone (ip,ip > 0,0; IPh,ip > IPh,0; IPh,ip > ip,0), supporting the ABH. Consequently, the authors speculated that ip and IPh boundaries are distinct when concerning predictions of the RBH and similar when considering the ABH. Following that line, they suggested that predictions concerning the ABH might result from lower level processes that chunk input for analysis and treat all breaks as identical, while prediction of the RBH could result from higher-level processes that attempt to align syntactic structure with a rich representation of prosodic structure.

Snedeker and Casserly’s approach to testing the ABH and the RBH is key to the understanding of the prosody-syntax interface as many of these predictions were never directly tested in previous studies, limiting inferences on the two hypotheses and their antecessors. Previous studies did, however, provide evidence for the role of the high boundary^(6,11,12)^ and for the relative size of the boundary^(8)^. Two studies that investigated the effect of a larger A boundary in contrast with no B boundary supported this specific prediction of the RBH^(10)^. However, there is no robust support for the RBH predictions when the B boundary is an ip. Snedeker and Casserly^(10)^ found the effect was reliable in subject analyses but not significant in items analyses. Clifton et al.^(7)^ tested several different syntactic structures and, although conclusions supported the RBH, some predictions (ip, ip > IPh, ip; 0, ip > IPh, ip) were not confirmed for all structures under investigation. Therefore, whether the relative size of the A boundary influences attachment when there is an ip boundary in B warrants further examination.

Similarly, previous studies failed to confirm ABH predictions that involve a larger B boundary^(2,8,10)^. Yet results were inconsistent when there was a larger A boundary (A > B). Snedeker and Casserly^(10)^ found that absolute size of a B boundary guided attachment when there was a larger A boundary while Carlson et al.^(8)^ did not find such an effect. One explanation for these differences may be that, although the sentences tested throughout the three studies were globally ambiguous, a manipulated version was compared to a “baseline” sentence. For sentences that have a low attachment preference, like those used by Carlson et al.^(8)^, there should be few low attachment responses on the 0, 0 conditions. Snedeker and Casserly^(10)^ found that in sentences with a preference for high attachment, the 0, 0 condition led to more high attachment, while Carlson et al.^(8)^ noted a preference for low attachment.

Another issue related to the prosody-syntax interface is that, to date, the predictions of ABH and RBH hypotheses have mainly been tested in English. Studies in other languages are needed to clarify whether this phenomenon is language-specific or is maintained regardless of the intonational systems and pitch accent distribution of each language. Lastly, age effects on the use of prosody on syntactic disambiguation for English-speaking children and adolescents have been reported^(12)^. Although children are sensitive to prosodic information, they use such information less effectively than adults to resolve syntactic ambiguation. Reports for Korean-speaking children seem to implicate a cross-linguistic developmental pattern, as children between four and five years old don’t seem to use prosody to disambiguate sentences^(13)^. The fact of Korean being a verb-final language might be related to that finding: the speculation is that children’s limited cognitive control prevents them from inhibiting misinterpretations or holding the noun phrases in memory to further analysis. It is critical that we examine developmental aspects of prosodic phrasing effects in a range of languages so that we can properly characterize any underlying processing principles governing prosodic phrasing.

### Cross-linguistic considerations of intonation and prosodic boundaries

Intonational structures differ across languages. English and Brazilian Portuguese are both stress-timed languages (basic rhythm is mainly determined by stressed syllables and the duration between two stressed syllables is equal). However, the location of stress in Brazilian Portuguese is less stress-timed and more predictable (the penultimate syllable is most often stressed), whereas stress location is not predictable in English. The acoustic parameters of stress are a complex mixture of pitch, intensity and duration. For both languages, stressed syllables have a higher pitch, are more intense, and are longer than unstressed syllables, though duration is more consistent in Brazilian Portuguese . There are further variations within dialects. Brazilian Portuguese, for instance, is not as strongly stress-timed as European Portuguese.

Although intonational systems and pitch accent distribution in sentences vary considerably among languages, the categorical characterization of null boundaries, ip, and IPh remains in many languages. For instance, French, English, and Brazilian Portuguese have different intonation but all exhibit robustness of ip and IPh characteristics^(14-16)^ which warrants the analysis of ABH and RBH as they apply to each. Differences in intonation may affect hypothesis predictions as they likely influence grouping of prosodic units. For example, it has been suggested that French speakers tend to equalize the size of the prosodic units they produce^(15)^. That is, French listeners can rely on the length of previous prosodic units in order to predict the length of the current one. This could theoretically constrain the types of expected syntactic constructions and interfere with the roles of prosodic boundaries on syntactic attachment. In word segmentation, listeners rely on the rhythmic structure of their language. If the effects of higher-level prosodic structure are similar to the effects of rhythmic structure, then the relationships across different levels of prosodic phrasing may be somewhat different across languages.

Most studies on prosodic boundaries and syntactic disambiguation in Brazilian Portuguese have examined reading. In general, these studies indicate that reading is possibly accompanied by the production of an implicit prosodic representation, supporting the Implicit Prosody Hypothesis^(17)^, and suggesting a prosodic boundary effect on disambiguation^(16)^. Studies analyzing the prosody-syntax interface in spoken language are limited in number and have not yet focused on hypotheses related to prosodic boundary strength (e.g., the RBH and the ABH) and its role in syntactic disambiguation. The scope of these studies is also limited, focusing only on whether prosodic boundaries affect comprehension of ambiguous sentences. It is critical to examine prosodic phrasing effects across languages in order to properly characterize underlying processing principles governing prosodic phrasing that properly guide clinical practices.

### Purpose and hypotheses

This study investigated how adults and children who are monolingual speakers of Brazilian Portuguese benefit from acoustic parameters of prosodic boundaries to disambiguate syntactically ambiguous sentences. The ABH and the RBH^(10)^ were tested in Brazilian Portuguese by varying the strength of the two boundaries in a sentence comprehension task (varying: F_0_, length of words preceding boundaries, and pauses following boundaries), allowing for examination of how relative and absolute boundary strengths influence disambiguation. Processing speed was also investigated through response time measures for each prosodic form to allow for comparison across groups.

It was hypothesized that, as in English, prosodic boundaries would have an effect on syntactic disambiguation in Brazilian Portuguese, given that intermediate phrases (ip) and intonational phrases (IPh) share similar roles in both languages. Even though children already show developed perceptual abilities with ips and IPhs by the age of eight, we anticipate that predictions involving strong prosody contrasts (IPh, 0 and 0, IPh) would be more likely to confirm than predictions with weak (ip, 0; 0, ip; IPh, ip; ip, IPh) or neutral prosody (identical prosodic boundaries on A and B (0, 0 and ip, ip), based on a possible stronger influence of acoustic salience on the prosody-syntax interchange for children. Furthermore, children would possibly be slower to process the ambiguities, as English-speaking adults are faster than adolescents (who, in turn, are faster than children) at resolving syntactic disambiguation^(12,18)^.

## METHODS

### Statement of ethics

This study was approved by institution under reference number 2015-0269 and comply with the guidelines for human studies and should include evidence that the research was conducted ethically in accordance with the World Medical Association Declaration of Helsinki. The adults and children (and their parents or guardians) have given their written informed consent and that the study protocol was approved by the institute’s committee on human research.

### Participants

Twenty-three adults (15 women and eight men) with mean age of 26;4 (±4;8) years and 15 children (seven girls and eight boys) with mean age of 9;9 (±1;3) years who were monolingual speakers of Brazilian Portuguese participated. The children’s data presented here served as control group in a previously published study^(19)^ that investigated the effects of prosodic boundaries on sentence comprehension in children with cochlear implants. All adults reported no history of language impairment, hearing impairment, or uncorrected visual impairment. Adult participants were undergraduate students or had higher educational degrees and were classified as middle or upper middle class according to the Brazilian Economic Classification Criterion questionnaire (CCEB - Critério de Classificação Econômica Brasil^(20)^. All children were tested on the vocabulary subtest of the ABFW Child Language Test^(21)^ (mean standard score = 93; SD = 2.5), and presented average nonverbal intelligence coefficient (IQ) measured by the Test of Non-Verbal Intelligence 4^(22)^ (mean standard score = 106; SD = 7.7) and no history of language impairment as reported by their parents, teachers, and school-based speech-language pathologists. All families of children were middle class based on a socio-economic questionnaire^(18)^ and all children passed a hearing screening at 25dB HL. The frequency discrimination (12.9 (±11.7) Hz and 8.5 (±7.3) Hz for children and adults, respectively) and gap detection thresholds (2.3 (±1.9) ms and 2.3 (±1.2) ms for children and adults, respectively) were sufficiently sensitive to detect the prosodic distinctions.

### Stimuli

The stimuli of the prosody experiment consisted of eight base sentences, each containing a prepositional phrase attachment ambiguity like those in the example (b) below.

- Formigas**_A_** e abelhas**_B_** com flores roxas estão na árvore.

Ants**_A_** and bees**_B_** with purple flowers are on the tree.

All sentences contained a noun phrase (NP) followed by a prepositional phrase (PP) and a verbal phrase (VP). All eight sentences had the same number of syllables for each NP, PP and VP . Prosodic boundaries were placed at A and B, respectively high and low boundaries (illustrated as a prosodic boundary pair A, B). The sentences were recorded in each of the eight prosodic boundary pairs: 0, 0; 0, ip; 0, IPh; ip, 0; ip, ip; ip, IPh; IPh, 0; IPh, ip.

All sentence stimuli were produced by one female native speaker of Brazilian Portuguese and were recorded using the Praat software a Philips Stereo Headphone SBC HP195 - M-Audio Mobile Pre USB (preamp and audio interface) and a B-5 Behringer Gold- Sputtered Diaphragm Studio Condenser Microphone, at the Phonetics Laboratory of Universidade Federal de Minas Gerais. The speaker was a linguist and produced each utterance in the most natural manner possible. Recordings focused on phonetic properties of boundaries characterized by changes in acoustic parameters, specifically duration and F_0_ changes immediately before the boundary and pauses immediately after the boundary. Therefore, intonational phrase (IPh) boundaries were accompanied by pauses of approximately 300 ms and the intermediate phrase (ip) boundary contained pauses of approximately 100 ms – null boundaries had no pauses.

### Statistical analysis of experimental manipulation of stimuli

In order to assure the experimental manipulation of stimuli was successful, the parameters related to prosodic boundaries (F_0_ and duration of nouns preceding boundaries and pauses after boundaries) were measured and statistically analyzed to ensure that consistent prosody was created across items in the same condition and that there was satisfactory contrast between different conditions. Table 2 shows the F_0_ and duration values (summed duration of the noun preceding the boundary and the pause after the noun) for each boundary type, independently of the boundary position (A or B).

**Table 2:** Mean (Standard Deviation) and [Range] of F0 Values of the Noun that Preceded the Prosodic Boundary (in Hz) and of the Summed Duration of the Noun and Pause that Preceded the Prosodic Boundary (in ms) According to Prosodic Boundary Type

**Boundary Type**	**Noun F_0_**	**Noun + Pause Duration**
0	228.6 (20.8)	857.7 (180.2)
	[193 – 317]	[557 – 1310]
ip	255.4 (27.6)	1557.2 (165.0)
	[210 – 343]	[1156 – 1901]
IPh	289.2 (42.2)	1829.1 (228.7)
	[241 – 447]	[1448 – 2430]

Caption: IPh = intonational boundary; ip = intermediate boundary; 0 = null boundary; F_0_ = fundamental frequency; Hz = Hertz; ms = millisecond

A one-way ANOVA revealed that the F_0_ of nouns preceding the three boundary types differed (*F*(2, 143) = 44,720, *p* < .001, *r* = .62). Post hoc tests with Bonferroni corrections indicated that the difference occurred at each comparison (all *p* < .001). The duration of the noun preceding the boundary summed with the duration of the boundary pause also differed across the three boundary types (*F*(2, 143) = 322,910, *p* < .001, r = .91). Post Hoc tests with Bonferroni corrections revealed that the differences occurred among all prosodic boundary types (all *p* < .001).

Table 3 displays F_0_ values of N1 and N2 (preceding boundaries A and B, respectively) according to boundary pairs. As expected, t-tests revealed that boundary pairs with identical prosodic boundaries in both A and B showed no differences in F_0_ of nouns 1 and 2 and an increased F_0_ in IPh when compared to ip, as well as ip when compared to null boundaries. The only exception was the 0, ip condition, in which the F_0_ of the two nouns did not differ. This is in line with arguments questioning whether F_0_ changes aid interpretation of syntactically ambiguous sentences in Brazilian Portuguese^(22)^. However, if this were true, the lack of an F_0_ difference should also be seen in the contrast between ip and null boundaries regardless of boundary position (see Table 3). The high variation of F_0_ values on the second noun could also explain the lack of significance. T-tests revealed no significant duration differences between boundaries A and B when the two prosodic pairs were identical (0, 0 and ip, ip). Statistically significant differences were found when the two prosodic boundaries were different on the pair – longer for IPh when compared to ip, and longer on ip when compared to null boundaries.

**Table 3:** Mean (Standard Deviation) and [Range] of F_0_ Values of Noun 1 and Noun 2 (in Hz) According to the Prosodic Boundary Pair

**Prosodic Boundary (A, B)**	**Noun 1 F_0_**	**Noun 2 F_0_**	**T-Test**
0, 0	219.4 (8.5)	207.1 (10.2)	*t*(7) = 2.944 *p* = .220, *r* = .55
	[210 – 237]	[193 - 221]	
0, ip	235.1 (14.9)	288.9 (41.7)	*t*(7) = -2.834, *p* = .250, *r* = .53
	[216 – 263]	[242 – 343]	
0, IPh	248.4 (32.0)	309.2 (64.8)	*t*(7) = -3.820, *p* = .007*, *r* = .68.
	[221 – 317]	[242 – 447]	
ip, 0	253.1 (16.3)	228.1 (12.3)	*t*(7) = 5.161, *p* = .001*, *r* = .79
	[227 – 271]	[205 – 248]	
ip, ip	256.6 (18.6)	237.6 (16.2)	*t*(7) = 2.225, *p* = .061, *r* = .41
	[232 – 288]	[210 – 255]	
ip, IPh	254.4 (14.0)	334.0 (23.3)	*t*(7) = -9.767, *p* < .001*, *r* = .93
	[237 – 278]	[304 – 385]	
IPh, 0	274.1 (14.7)	233.2 (14.2)	*t*(7) = 5.461, *p* = .001*, *r* = .81
	[254 – 292]	[212 – 251]	
IPh, ip	266.0 (17.2)	241.6 (21.3)	*t*(7) = 2.804, *p* = .026*, *r* = .53
	[241 – 284]	[221 – 283]	

* Significant difference at p < 0.05.

Caption: IPh = intonational boundary; ip = intermediate boundary; 0 = null boundary; F_0_ = fundamental frequency; Hz = Hertz

Sixteen unambiguous sentences were mixed with the target experimental sentences in order to create two contrasting prosodies and decrease awareness of the target manipulation. Eight filler sentences contained a predicate attachment, like in (c), and eight contained a reflexive assignment, like in (d). These filler sentences were previously used in studies that did not investigate prosody^(19,23-25)^. New recordings of the original sentences were made, focusing on stress manipulations. For example, in sentences such as (c), stress was alternately placed on noun 1 (N1) (horse) or noun 2 (N2) (barn) and the same was done for type (d) sentences. There were no pauses after N1 or N2.

- O cavalo **_N1_** atrás do celeiro **_N2_** é laranja.

The horse **_N1_** behind the barn **_N2_** is orange.

- A mãe **_N1_** na frente da avó **_N2_** está se lavando.

The mom **_N1_** behind the grandma **_N2_** is washing herself.

**Figure 1:**
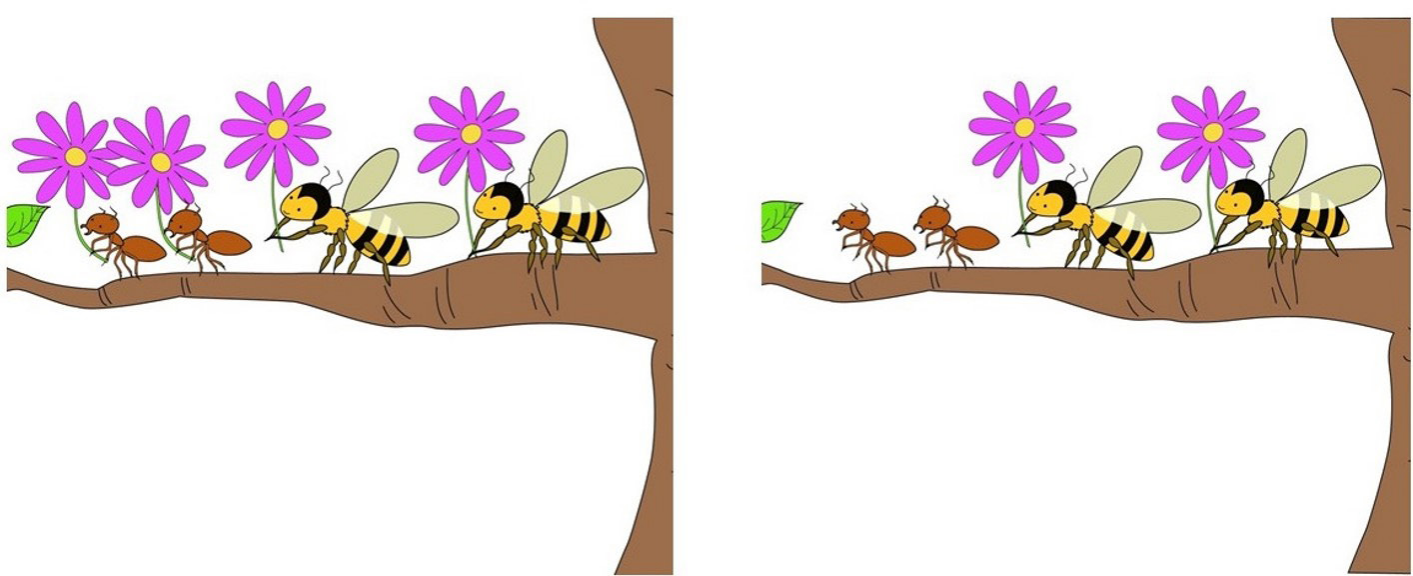
Visual stimuli for the target sentence *Formigas e abelhas com flores roxas estão na árvore.* (Ants and bees with purple flowers are on the tree)

A pair of visual stimuli (pictures) was created for each sentence. For the target sentences, one picture reflected the low attachment and the other represented the high attachment interpretation of the sentence. Positioning of the pictures on the left and right halves of the computer screen was assigned randomly by the E-Prime software. Below (Figure 1) is an example of visual stimuli for the target sentence *Ants and bees with purple flowers are on the tree* (Formigas e abelhas com flores roxas estão na árvore). The picture on the right reflects a high attachment response, while the picture on the left reflects a low attachment response. All pictures were drawn by a single artist.

### Procedure

E-Prime 2.0 software was used for stimuli presentation and data collection. Participants were individually tested in a quiet room where they were seated in front of the computer with a serial response box (SRBOX) and high definition speakers placed before them. All stimuli were presented at the most comfortable loudness level on an individual basis.

In each trial, participants heard a sentence one time. Immediately after the offset of the sentence, two pictures with the two possible interpretations appeared on the screen. For target stimuli, such as the sentence *Ants and bees with purple flowers are on the tree,* one option showed both ants with purple flowers and bees with purple flowers on a tree, while the other showed ants without purple flowers and bees with purple flowers on a tree. With the pictures still visible, after an interval of 2000 ms, the sentence was repeated. Participants pressed a button on the SRBOX to indicate their selection of interpretation. To select the figure on the left, they needed to press 1, the leftmost button on the SRBOX. To select the figure on the right, they needed to press 5, the rightmost button on the SRBOX. Responses were accepted no earlier than the offset of the second sentence stimuli. Inter-trial intervals had a duration of 1000 ms. A practice session containing 10 trials preceded the experiment.

Each participant heard 64 stimuli and 32 filler stimuli. The stimuli were presented to participants in four blocks. Block A referred to strong prosody contrasts, containing trials with one intonational phrase and one null boundary (IPh, 0 and 0, IPh). Block B consisted of trials reflecting neutral prosody with identical prosodic boundaries on A and B (0, 0 and ip, ip). Block C referred to trials with weak prosody contrasts, with trials containing intermediate phrase and null boundary (ip, 0 and 0, ip). Block D contained trials with two prosodic boundaries (IPh, ip and ip, IPh). Blocks were randomly assigned. Within each block, the stimuli were presented in a random order to avoid length, order, or familiarization effects. The average length of time to complete the experiment was 30 minutes.

### Statistical methods

The prosody task consisted of a binomial experiment, as there were only two mutually exclusive possible responses. Therefore, the data was analyzed according to sample proportions (p̂): the proportion of high attachment responses was calculated for each of the eight prosodic forms within each participant. These numbers were analyzed using mixed repeated-measures and one-way ANOVAs and contrasts were applied where necessary. Planned between-group comparisons were carried out using independent-means t-test for each prosodic form with Bonferroni corrections. The specific predictions of the Absolute Boundary Hypothesis (ABH) and the Relative Boundary Hypothesis (RBH)^(10)^ were tested using planned dependent-means t-tests with Bonferroni corrections.

Response times were calculated from the offset of the second time the target sentence was played to the moment the response button on the serial response box was pressed. Only response times for high attachment responses were considered. Outliers were removed to calculate the mean response times and perform the inferential analyses. Outliers were identified as response times that were more than a 1.5 interquartile range (distance between the first and third quartiles) below the first quartile (minor outliers) or above the third quartile (major outliers). No minor outliers were identified – no response times below 200ms were found in this sample. A total of 12 major outliers (0.49% of all data) were identified (four in the group of adults, eight in the group of children). Mixed repeated-measures ANOVA were calculated and contrasts applied where necessary. Planned between-group comparisons were carried out using independent-means t-test with Bonferroni corrections for each prosodic form.

## RESULTS

### Attachment

The proportions of high attachment responses for each of the eight prosodic sentence types were calculated for each group (see Table 4). Sentences with no boundaries (0,0) had the most high attachment interpretations for both groups.

**Table 4:** Mean (Standard Error) of the Proportion of High Attachment Response According to Prosodic Boundary Type and Group. Between-group Comparisons are Indicated by Independent t-tests with Bonferroni Corrections

**Prosodic Boundary (A, B)**	**Children**	**Adults**	**T-Test**
0, 0	.94 (.03)	.88 (.04)	*t*(36) = -1,250, *p* = .872, *r* = .20
0, ip	.48 (.09)	.46 (.07)	*t*(36) = -,162, *p* = 1.00, *r* = .03
0, IPh	.68 (.06)	.59 (.06)	*t*(36) = -,944, *p* = 1.000, *r* = .16
ip, 0	.44 (.08)	.57 (.06)	*t*(36) = 1,256, *p* = .872, *r* = .20
ip, ip	.43 (.07)	.56 (.05)	*t*(36) = 1,560, *p* = .512, *r* = .25
ip, IPh	.47 (.10)	.69 (.05)	*t*(36) = 2,350, *p* = .096, *r* = .36
IPh, 0	.41 (.07)	.57 (.07)	*t*(36) = 1,561, *p* = .512, *r* = .25
IPh, ip	.39 (.08)	.65 (.05)	*t*(36) = 2,888, *p* = .024^*^, *r* = .43

* Significant difference at p < 0.05.

Caption: IPh = intonational boundary; ip = intermediate boundary; 0 = null boundary

Mixed design ANOVA revealed a significant effect for prosodic type *F*(7,252) = 12,136, *p* < .001, η2 = .252, indicating that interpretation of the target sentences varied according to their prosodic forms. Contrasts revealed that sentences with two null boundaries received the highest number of high attachment responses (all *p <* .05). There was also a significant interaction between prosodic type and age group *F*(7,252) = 2,509, *p* = .016, η2 = .065, indicating that children and adults differed in how they used prosodic forms to disambiguate sentences. There was a trend for a group effect, *F*(1,36) = 3,645, *p* = .064, η2 = .092, which possibly missed significance due to the small sample size. Planned pairwise independent samples t-tests with Bonferroni corrections comparing the two groups for proportion of high attachment interpretations according to prosodic type were calculated (Table 4). Results indicated that children and adults differed in the proportion of high attachment interpretations in the IPh, ip condition, with adults showing more high attachment responses than children.

To investigate the specific predictions of RBH and ABH, planned pairwise comparisons were conducted for each group. Results of RBH are displayed in Table 5. Only four of the five hypotheses for the current study were confirmed. Regarding specific predictions of RBH, only two were confirmed for children and adults. When a low boundary (null boundary in position B) was absent, the size of the high boundary (position A) influenced attachment. Thus, a larger boundary in A led to more high attachments than a smaller boundary, confirming the first two specific predictions of the RBH. Contrastively, when there was a low ip boundary (ip in B), the relative size of the high boundary (A) did not influence attachment in the way predicted by the RBH. Sentences with a high null boundary and a low ip led to less high attachment responses than sentences with a high ip or IPh, in contrast to what the RBH predicted. Furthermore, the number of high attachment did not differ from sentences with two ips and sentences with a smaller (0) or bigger (IPh) high boundary, rejecting, in this case, the prediction of the RBH that the size of the high boundary would guide attachment.

**Table 5:** Unique Predictions of ABH and RBH Tested in This Study According to Prosodic Form, Group, and Hypotheses of the Current Study

	**Prosody**	**Prediction**	**Hypotheses and Findings for Children**	**Hypotheses and Findings for Adults**
ABH	Equal boundaries	0, 0 < ip, ip	Not expected	Expected
			*t*(14) = 6,082, *p* < .001*^A^, *r* = .85	*t*(22) = 5,833, *p* < .001*^A^, *r* = . 84
	Larger high boundary	IPh, 0 < IPh, ip	Expected	Expected
			*t*(14) =, 349, *p* = .366, *r* = .09	*t*(22) = -1,141, *p* = .266, *r* = . 29
		ip, 0 < IPh, ip	**Not expected**	Expected
			*t*(14) =, 771, *p* = .226, *r* = .20	t(22) = -1,189, *p* = .247, *r* = . 30
	Larger low boundary	0, ip < 0, IPh	**Expected**	Not expected
			*t*(14) = -2,195, *p* = .023*, *r* = .51	*t*(22) = -2,893, *p* = .008*, *r* = . 61
		0, ip < ip, IPh	**Not expected**	Not expected
			*t*(14) =, 125, *p* = .451, *r* = .03	*t*(22) = -3,062, *p* < .006*, *r* = . 63
RBH	No low boundary	IPh, 0 < 0, 0	**Expected**	**Expected**
			*t*(14) = -7,400, *p* < .001^*^, *r* = .89	*t*(22) = -4,208, *p* < .001*, *r* = . 75
		ip, 0 < 0, 0	Not expected	**Expected**
			*t*(14) = -5,563, *p* < .001*, *r* = .83	*t*(22) = -3,920, *p* = .001*, *r* = . 72
	Low ip boundary	IPh, ip < 0, ip	**Not expected**	Not expected
			*t*(14) = -,731, *p* = .238, *r* = .19	*t*(22) = 2,393, *p* = .026*^A^, *r* = . 54
		ip, ip <0, ip	**Not expected**	**Not expected**
			*t*(14) = -,437, *p* = .334, *r* = .12	t(22) = 1,098, *p* = .284, *r* = . 28
		IPh, ip < ip, ip	**Not expected**	**Not expected**
			*t*(14) = -,857, *p* = .203, *r* = .22	*t*(22) = 1,854, *p* = .077, *r* = . 44

* Significant difference at p < 0.05.

Caption: ABH = Absolute Boundary Hypothesis; IPh = intonational boundary; ip = intermediate boundary; 0 = null boundary

A significant difference with inverse relationship of prediction. Confirmed hypotheses are in bold.

Planned pairwise comparisons were also carried out to investigate the specific predictions of ABH in each group and results are also displayed in Table 5. None of the hypotheses for the current study were confirmed for adults and only three of the five were confirmed for children. The ABH predicts that only the absolute size of the low boundary affects attachment. Thus, regarding specific predictions of the ABH, under the condition of two equal boundaries, sentences that have larger boundaries would lead to more high attachment responses than those with smaller boundaries. This prediction was not confirmed for either group, as sentences with two null boundaries had more high attachment responses than sentences with two ips. Additionally, according to the ABH, the absolute value of the low boundary should command attachment regardless of the size of the high boundary. In other words, the larger the low boundary the higher the proportion of high attachment responses regardless of the size of the high boundary. However, results showed that when there was a larger high boundary (i.e., A was bigger than B), the absolute size of B did not influence attachment in Brazilian Portuguese-speaking adults. Different outcomes for children and adults were observed when the low boundary was larger than the high boundary. For children, the absolute value of the boundary at B affected interpretation as predicted by the ABH only when there was no low boundary. For adults, however, both predictions involving a larger low boundary were confirmed, regardless of whether the high boundary was an ip or a null boundary.

In sum, the same two predictions of the RBH (0, 0 > IPh, 0; 0, 0 > ip, 0) were confirmed for both groups, indicating that the RBH is partially confirmed for children and adults who speak Brazilian Portuguese. In contrast, the ABH testing had different outcomes for the two groups. Two specific predictions of ABH (0, ip < 0, IPh; 0, ip < ip, IPh) were confirmed for adults. However, only one of the predictions (0,ip < 0,IPh) was confirmed for children. One prediction (ip, ip < 0, 0) of the ABH had an inverse outcome for both children and adults: a bigger boundary led to less high attachment responses than a sentence with no boundary.

### Response time

Table 6 illustrates the mean response times (in ms) according to prosodic form and group. Overall, children exhibited longer response times than adults and had greater variation across prosody types. Mixed design ANOVA revealed a significant effect for prosodic type *F*(7,154) = 2,145, *p* = .042, η2 = .089, indicating that response times varied according to prosodic forms. There was a significant interaction between prosodic type and group *F*(7,154) = 2,151, *p* = .042, η2 = .089, indicating that children and adults differed in how fast they gave high attachment responses according to the examined prosodic types. Eight planned pairwise comparisons with independent samples t-tests and Bonferroni corrections for response times for high attachment interpretations were calculated according to prosodic type. Results indicated that children were significantly slower to produce a high attachment response than adults in all but the 0,0 prosodic type (see Table 6). The Bonferroni corrections applied to the comparisons could have inflated the type II error, explaining the results for the neutral 0,0 condition. This could be a reflection of the high variability exhibited by children in this specific prosodic condition.

**Table 6:** Mean (Standard Error) of Response Times (ms) to High Attachment Responses According to Prosodic Boundary Type and Group. Between-group Comparisons are Indicated by Independent t-tests with Bonferroni Corrections

**Prosodic Boundary (A, B)**	**Adults**	**Children**	**T-Test**
0, 0	401 (26)	2909 (1381)	*t*(35) = -2,348, *p* = .200, *r* = .37
0, ip	449 (47)	1321 (207)	*t*(28) = -4,915, *p* < .001^*^, *r* = .68
0, IPh	521 (33)	1638 (253)	*t*(34) = -5,159, *p* < .001*, *r* = .66
ip, 0	390 (24)	1738 (450)	*t*(30) = -3,638, *p* = .008*, *r* = .55
ip, ip	441 (33)	1788 (416)	*t*(33) = -4,487, *p* < .001*, *r* = .62
ip, IPh	375 (29)	1337 (312)	*t*(34) = -4,090, *p* < .001*, *r* = .57
IPh, 0	551 (58)	2732 (656)	*t*(32) = -4,224, *p* < .001*, *r* = .60
IPh, ip	446 (23)	1536 (322)	*t*(32) = -4,918, *p* < .001*, *r* = .66

* Significant differences at p < 0.05.

## DISCUSSION

This study examined prosodic boundary effects in Brazilian Portuguese by analyzing how children and adults benefit from prosodic boundaries to disambiguate syntactically ambiguous sentences. The Relative Boundary Hypothesis and the Absolute Boundary Hypothesis^(10)^ were contrasted. To date, previous studies in Brazilian Portuguese had only examined whether a boundary affects interpretation (mainly in reading), but not prosodic boundary strength effects on syntactic disambiguation. There is a need for studies in languages other than English that explore prosodic boundary effects to determine whether effects are language-specific and whether they play a similar role in syntactic processing in languages with different prosodic specificities. Novel information on developmental differences in attachment of prosodic boundaries was collected.

### Relative Boundary Hypothesis

Only four of the five hypotheses for the current study were confirmed, suggesting that acoustic salience does not completely guide the prosody-syntax interface for children and that cross-linguistic differences are present for adults. Two predictions of the RBH were confirmed for adults and children, suggesting that both groups perceive and use the relative size of the boundaries. Without a low boundary, the relative size of the boundary in a higher position affected attachment; likewise, the presence of a larger high boundary discouraged high attachment as occurred with English-speaking adults^(10)^. Therefore, it is likely that the relative size of the high boundary has an effect on syntactic disambiguation, regardless of age, when there is no low boundary as predicted by the RBH.

The RBH did not hold true when a sentence had a low intermediate phrase (ip) boundary. The presence of a low ip did not allow the size of the high boundary to influence attachment for Brazilian Portuguese-speakers, regardless of age. Snedeker and Casserly^(10)^ only found significant effects in the subject analyses but not in the items analyses. Clifton et al.^(7)^ tested several different syntactic structures and, although they found an overall support for RBH, these predictions (ip, ip > IPh, ip; 0,ip > IPh, ip) did not hold true for –ly adverbs.

Word stress may explain the lack of effect of the low ip. Schafer et al.^(26)^ suggested that the largest prosodic boundary generally corresponded to the largest syntactic boundary, as listeners used the relative size of boundaries to disambiguate sentences. Adult listeners resolved sentence ambiguities utilizing unidentified informational cues other than the prosodic boundaries. The authors speculated that stress patterns could have provided the necessary information, as prosodic boundaries often follow the most important accent in a phrase. Thus, a boundary could increase the salience of the preceding word, increasing the likelihood of attachment to that word. In the current study, the presence of an IPh in the prosodic boundary pair (IPh, ip) increased stress on the first noun (as shown by F_0_ and duration analyses in Table 2 and 3, respectively), which might have forced attachment to a higher position. In contrast, Carlson et al.^(8)^ observed a low attachment bias, where placement of stress may have overcome the effects of prosodic boundaries – at least in sentences with a high attachment preference and a low ip. For Brazilian Portuguese-speaking adults, one of the RBH predictions involving a low ip boundary also showed a reversed outcome. A larger high than low boundary (IPh, ip) led to more high attachment responses, suggesting that an IPh in a high position discouraged low attachment.

Snedeker and Casserly^(10)^ suggested that IPh and ip have distinct roles in the predictions of the RBH, but similar roles in predictions of the ABH. This does not explain the RBH findings for Brazilian Portuguese. That is, we should have seen differences in proportion of high attachment between (IPh, ip) and (ip, ip) sentences, which did not occur. For English speakers, previous studies have confirmed the prediction that sentences with (IPh, ip) led to less high attachment interpretations than sentences with (ip, ip)^(2,8,10)^. However, this is a single prediction (out of two) that allows such comparison. The only other RBH prediction that would support the distinct roles of IPh and ip is the IPh, ip < 0, ip. If IPhs and ips have distinct roles, the presence of an IPh in A would accordingly favor low attachment in the (IPh, ip) sentence. However, this could occur even if IPh and ip have similar roles (resulting in no preference in attachment). This prediction would still lead to fewer high attachment responses than a (0, ip) sentence (ip in B favors high attachment under both hypotheses). In sum, the RBH does not explain the effects of prosodic boundaries on syntactic disambiguation as previously suggested, and it seems to only account for limited instances of findings.

### Absolute Boundary Hypothesis

None of the hypotheses for the current study were confirmed for adults on the ABH, making it clear that cross-linguistic differences exist on the prosody-syntax interface between English and Brazilian Portuguese. Only three of the five hypotheses were confirmed for children, once again suggesting that acoustic salience does not completely guide the prosody-syntax interface for children.

Two of the five predictions of the ABH were confirmed for adults, whereas only one was confirmed for children. For adults, both predictions involving a larger low boundary were confirmed - regardless of whether the high boundary was an ip or a null boundary. This contrasts with previous findings for English-speaking adults^(8,10)^ that failed to confirm these predictions of the ABH. The contrast between two boundary types and the position of boundaries may have different weights cross-linguistically. When the high boundary was bigger (more acoustically salient) than the low boundary (A > B), the contrast between ip and 0 and the predictions IPh, 0 < IPh, ip and ip, 0 < IPh, ip were confirmed only for English^(10)^. When the low boundary was bigger (B > A), the contrast between IPh and ip and the two predictions 0, ip < 0, IPh and 0, ip < ip, IPh were confirmed only in Brazilian Portuguese. In Brazilian Portuguese, boundaries in B may be more relevant than boundaries in A, and IPhs may be more relevant than ips. For the children, however, the absolute value of the low boundary affected interpretation only when there was no high boundary, providing partial support for the ABH.

No predictions involving weak (ip and 0) acoustic contrasts were confirmed in Brazilian Portuguese, while findings in English have been mixed. Snedeker and Casserly^(10)^ found support for ABH hypotheses that depended on weak acoustic contrasts, whereas Carlson et al.^(8)^ found no support for the ABH. Overall, the influence of boundary salience on predictions of ABH is only partially supported for Brazilian Portuguese. Yet the joint operation of acoustic salience and how prosodic boundaries govern syntactic disambiguation is not straightforward. Additional components, such as linguistic variability, processing, and executive function demands may interfere with this process.

When there was a larger high boundary (A > B), the size of the low boundary did not influence attachment for all Brazilian Portuguese-speakers, consistent with the ABH. Results from previous studies in English were inconsistent. Snedeker and Casserly^(10)^ found the absolute size of a low boundary guided attachment when there was a larger high boundary while Carlson et al.^(8)^ did not. Several factors could explain these discrepancies. Although the sentences tested in the three studies were globally ambiguous, a manipulated version is being compared to a “baseline” sentence. For sentences with a low attachment preference, there should be few low attachment responses on the (0, 0) conditions - the reverse is true for sentences with a high attachment preference. Like the current study, Snedeker and Casserly^(10)^ found that in sentences with a preference for high attachment, the (0, 0) condition led to more high than low attachment. Carlson et al.^(8)^ found preference for low attachment. Although these findings do not explain the cross-linguistic differences, it might explain the differences observed between the two studies in English. The proportion of high attachment in the study by Carlson et al.^(8)^ on the two prosodic forms (IPh, 0, IPh, ip) were both .15, whereas in the study by Snedeker and Casserly^(10)^ they were around .45 and .70, respectively. The low attachment preference for the sentences in the study by Carlson et al.^(8)^ created a floor effect that concealed differences.

Task type might also account for some of the observed differences among studies. Carlson et al.^(8)^ used a timed unacceptability judgment task, in which participants were asked questions only when a sentence was identified as acceptable This method may have created an increased awareness of manipulations within task stimuli. Both Snedeker and Casserly’s^(10)^ study and the current study used a sentence comprehension task with two visual response options. The lack of unambiguous filler sentences that included the same characters or objects as target ambiguous sentences - and forced response to each of the two pictures (high and low attachment) - could have created an overall bias in current and previous studies. The type of instruction provided could also have influenced findings. In Snedeker and Casserly^(10)^ and in the current study, the participants were told that the meaning of the sentence could change depending on *how* the sentence was said. This was not clearly stated to the participants in previous studies.

The inverse of ABH attachment predictions when comparing (0, 0) and (ip, ip) occurred in all Brazilian Portuguese speakers. Yet Snedeker and Casserly^(10)^ found support for the 0, 0 < ip, ip ABH prediction with English speaking adults. Perhaps participants in the current study treated a (0, 0) sentence as *natural* and the (ip, ip) as an *unnatural* manipulated version in which a high boundary discouraged high attachment regardless of the presence of a low boundary (the reverse of the ABH). However, for adults in the current study, the presence of a high ip did not discourage high attachment when the low boundary was larger. Another possibility is that in sentences with two identical boundaries (ip, ip), the relevance of the high boundary was considered stronger. (0, 0) had more high attachment responses than (ip, ip); the absence of a high boundary joined the two constituents favoring high attachment. However, this high boundary bias did not hold true for sentences with two different boundaries. High attachment responses to the unmanipulated version could have also occurred because of a high attachment preference in Brazilian Portuguese.

Clifton et al.^(7)^ suggested that parallel analysis could explain the high number of high attachment on the *prosodically uninformative* sentences (0, 0 and ip, ip). Two relatively simple noun phrases (NPs) would be adjoined, leading to a high attachment interpretation. However, in a low attachment interpretation, one constituent might be substantially more complex than the other, disrupting the parallelism between NPs. Parallel analysis could be an explanation for the attachment preference under the neutral boundaries condition of the current study. Parallel analysis may also explain the overall high attachment preference for adults, as a low attachment preference would create an unbalanced distribution of constituents. Clifton et al.^(7)^ expected this to also interfere in attachment in the (ip,ip) condition, which did not occur in the current study. Children did not exhibit a high attachment preference for (ip, ip) sentences and such preference was not remarkable in adults. Indeed, these conditions had an outcome that was the reverse of what has been found in English: in Brazilian Portuguese, (ip, ip) led to less high attachment than (0, 0) sentences. How the parallel analysis proposal would interact with prosodic boundary information warrants further consideration of why, when, and whether one would overcome the other.

The 0, 0 < ip, ip was the only prediction in which both prosodic forms (0, 0 and ip, ip) were tested in the same block and compared to English, resulting in a reversed outcome (ip, ip < 0, 0) for all participants. This reversion may have been caused by the executive function demands of the within subject design of the current study, as suggested by Snedeker and Yuan^(18)^. In a within subject design, the response of the current trial is affected by the response of the previous trial, but the presence of filler sentences serves to attenuate such effect. In the current study, the potential executive function demands (such as inhibiting the response to previous trials) did not reverse the predictions in all prosodic forms, indicating that some conditions were more challenging than others. One of the RBH predictions also lead to the reverse outcome for adults (more high attachment for IPh, ip than 0, ip) and these two prosodic forms were not tested in the same block, obviating the within subject design effect. Therefore, it is possible that these reversed outcomes also involve the pure prosody-syntax interface.

### Developmental considerations

The results of the present study indicate that 8-12 year-old Brazilian Portuguese-speaking children differ from adults in the way they use prosodic boundaries to disambiguate sentences. Furthermore, results indicate that acoustic salience of boundaries dot not explain the difference between children and adults in using prosody information to disambiguate sentences.

Research (largely on English) has shown that the IPh, 0 condition results in less high attachment than the 0, IPh condition^(1,27)^. However, that finding was not obtained in the present study for adults (although it was for children). Besides pointing to cross-linguistic differences between English and Brazilian Portuguese, these finding also points to developmental aspects of prosodic effects of sentence processing in Brazilian Portuguese. The use of conjoined NPs in the current study do not explain the findings for adults since other studies (including^(1)^) have also used these types of stimuli. Perhaps the length of the NPs involved could have had an effect for the adults’ findings. Clifton et al.^(7)^ showed that prosodic boundary effects were modulated by phrase length. The length of the NPS in the current study could have affected the lack of difference in proportion of high attachment between the IPh, 0 and the 0, IPh condition for the adults. It is possible that the phrase length modulation occurs differently for children.

For children, only predictions of RBH and ABH that involved sentences with no low or no high boundaries were confirmed. When predictions involved two boundaries, children were not able to benefit from prosodic boundary information to select the site of attachment. RBH predictions involving a low ip support the hypothesis that only one boundary is relevant, for children. When the low boundary is an ip, the high boundaries were treated similarly, regardless of type. Neither the type nor the dimension of acoustic characteristics were sufficient as standalone features of prosodic boundaries for children to disambiguate sentences. It is unlikely that the size of a boundary alone was the reason for these findings, as IPh and ip involved more extreme acoustical changes than ip and 0. The findings for the specific predictions of ABH also support the one-boundary explanation for children, as only the 0, ip < 0, IPh prediction was confirmed.

Exclusive support of single boundary predictions suggests that children are unable to benefit from multiple sources of prosodic boundary information. A speculation is that working memory may be involved, as it is more demanding to retain information about two different boundaries and compare them, than it is to consider only one boundary. The process of binding (between boundaries and phrase structure) and release from binding described in recent models of working memory^(28)^ may well apply. In the current study, no measures of working memory or executive functions were included. However, the increase in memory capacity across development is accompanied by an increase in processing speed across a wide range of tasks^(29)^. Therefore, detailed response time analyses could aid in explaining some developmental differences.

Although some differences were observed in how prosodic boundaries influenced attachment of ambiguous sentences in children and adults, processing speed revealed substantial differences, possibly reflecting developmental differences in timing of prosodic effects (in addition to limitations in working memory). Adults were notably faster than children in arriving at a high attachment response, responding on average almost one and a half seconds quicker than children. Outside of limitations in working memory, this difference may reflect children’s need to create and utilize strategies to resolve ambiguity. They may not be able to automatically resolve syntactically ambiguous sentences, which may reflect weaker cognitive control – an executive function that is related to the capacity to detect and resolve conflicts between differing representations^(30)^. This aspect of executive function emerges between the ages of five and 11 years. Eye-tracking studies suggest that the effect of prosody on syntactic disambiguation occurs at different processing-time points in English-speaking children, adolescents, and adults^(12,18)^.

Some limitations of the current study include the lack of cognitive measures, such as executive function abilities, that could have played a role in the interaction between prosody and syntax. The use of offline measures provided no information on the routes that took the participants to choose their responses and possible factors underlying their decision. Although data from children and adults provide insights on developmental aspects on the prosody-syntax interplay, the absence of data on adolescents does not provide complete developmental picture.

The role of prosodic boundaries on syntactic disambiguation within and across languages is far from being well understood. Suggestions for future studies include the application of online measures, such as eye-tracking, that would indicate which linguistic or suprasegmental (or even cognitive) components underlie this difference in processing speed. Future studies should include adolescents to provide a more complete developmental picture, as well as additional syntactic structures. The manipulation of working memory and other executive function demands could provide the means to determine their potential roles in listeners’ abilities to process syntax and prosody simultaneously. Moreover, the use of online measures, such as ERPs or eye-tracking, would provide continuous data and yield a time course of the interface between prosodic boundaries and syntactic disambiguation. Online studies would permit an examination of which specific information aids in disambiguation and when that information becomes available.

## CONCLUSION

Neither the ABH nor the RBH explain how prosodic boundaries influence attachment in Brazilian Portuguese-speaking children and adults. The relationship between position and size of boundaries in resolution of syntactic ambiguities is not straightforward and cannot be fully explained by their predictions. This relationship may be influenced by additional factors such as attachment preferences, cross-linguistic differences, and aspects of working memory demands. This study revealed that the ABH and RBH are not consistent cross-linguistically, as different predictions were confirmed for Brazilian Portuguese, contrasting with previous findings for English. Acoustic salience does not guide the prosody-syntax interface for children. Furthermore, children differed from adults in the way they used prosodic boundaries to disambiguate sentences and there were notable differences in response time, indicating that the interplay between prosody and syntax should be considered differently for each group when guiding clinical practices.

## References

[1] Deniz ND, Fodor JD (2020). Timing of syntactic and rhythmic effects on ambiguity resolution in turkish: a phoneme restoration study. Lang Speech.

[2] Carvalho A, Christophe A, Dautriche I, Lin I (2017). Phrasal prosody constrains syntactic analysis in toddlers. Cognition.

[3] Kolberg L, de Carvalho A, Babineau M, Havron N, Fiévet A-C, Abaurre B (2021). “The tiger is hitting! the duck too!” 3-year-olds can use prosodic information to constrain their interpretation of ellipsis. Cognition.

[4] Caccia M, Lorusso ML (2019). When prosody meets syntax: the processing of the syntax-prosody interface in children with developmental dyslexia and developmental language disorder. Lingua.

[5] Dinçtopal Deniz N, Fodor JD (2017). Phrase lengths and the perceived informativeness of prosodic cues in Turkish. Lang Speech.

[6] Watson D, Gibson E (2005). Intonational phrasing and constituency in language production and comprehension. Stud Linguist.

[7] Clifton C, Carlson K, Frazier L (2002). Informative prosodic boundaries. Lang Speech.

[8] Carlson K, Clifton C, Frazier L (2001). Prosodic boundaries in adjunct attachment. J Mem Lang.

[9] Beckman ME, Hirschberg J (1994). The ToBI annotation conventions..

[10] Snedeker J, Casserly E (2010). Is it all relative? Effects of prosodic boundaries on the comprehension and production of attachment ambiguities. Lang Cogn Process.

[11] Mitterer H, Kim S, Cho T (2021). The role of segmental information in syntactic processing through the syntax–prosody interface. Lang Speech.

[12] Diehl JJ, Friedberg C, Paul R, Snedeker J (2015). The use of prosody during syntactic processing in children and adolescents with autism spectrum disorders. Dev Psychopathol.

[13] Choi Y, Trueswell JC (2010). Children’s (in)ability to recover from garden paths in a verb-final language: evidence for developing control in sentence processing. J Exp Child Psychol.

[14] Snedeker J, Trueswell J (2003). Using prosody to avoid ambiguity: effects of speaker awareness and referential context. J Mem Lang.

[15] Monnin P, Grosjean F (1993). Les structures de performance en français: caractérisation et prédiction. Annee Psychol.

[16] Magalhães J, Maia M (2017). Pistas prosódicas implícitas na resolução de ambigüidades sintáticas: um caso de adjunção de atributos. Rev ABRALIN..

[17] Fodor JD (2002). Prosodic disambiguation in silent reading..

[18] Snedeker J, Yuan S (2008). Effects of prosodic and lexical constraints on parsing in young children (and adults). J Mem Lang.

[19] Fortunato-Tavares T, Schwartz R, Marton K, de Andrade C, Houston D (2018). Prosodic boundary effects on syntactic disambiguation in children with cochlear implants. J Speech Lang Hear Res Online..

[20] ABEP: Associação Brasileira de Empresas de Pesquisa (2021). Critério Brasil.

[21] Andrade CRF, Lopes DMB, Fernandes FDM, Wertzner HF (2011). ABFW: teste de linguagem infantil nas áreas de fonologia, vocabulário, fluência e pragmática..

[22] Brown L, Sherbenou RJ, Johnsen SK (2021). TONI4 Test of Nonverbal Intelligence Fourth Edition.

[23] Fortunato-Tavares T, Andrade CRF, Befi-Lopes D, Limongi SO, Fernandes FDM, Schwartz RG (2015). Syntactic comprehension and working memory in children with specific language impairment, autism or Down syndrome. Clin Linguist Phon.

[24] Fortunato-Tavares T, Andrade CRF, Befi-Lopes DM, Hestvik A, Epstein B, Tornyova L (2012). Syntactic structural assignment in Brazilian Portuguese-Speaking Children with specific language impairment. J Speech Lang Hear Res.

[25] Fortunato-Tavares T, Howell P, Schwartz RG, Andrade CRF (2017). Children who stutter exchange linguistic accuracy for processing speed in sentence comprehension. Appl Psycholinguist.

[26] Schafer A, Carlson K, Clifton H, Frazier L (2000). Focus and the interpretation of pitch accent: disambiguating embedded questions. Lang Speech.

[27] Frazier L, Carlson K, Clifton C (2006). Prosodic phrasing is central to language comprehension. Trends Cogn Sci.

[28] Shepherdson P, Oberauer K (2018). Pruning representations in a distributed model of working memory: a mechanism for refreshing and removal?. Ann N Y Acad Sci.

[29] Kail R, Park Y-S (1994). Processing time, articulation time, and memory span. J Exp Child Psychol.

[30] Huang YT, Hollister E (2019). Developmental parsing and linguistic knowledge: reexamining the role of cognitive control in the kindergarten path effect. J Exp Child Psychol.

